# Comparative Cellular Toxicity of Hydrophilic and Hydrophobic Microcystins on Caco-2 Cells

**DOI:** 10.3390/toxins4111008

**Published:** 2012-10-25

**Authors:** Pia S. M. Vesterkvist, Julia O. Misiorek, Lisa E. M. Spoof, Diana M. Toivola, Jussi A. O. Meriluoto

**Affiliations:** 1 Biochemistry, Department of Biosciences, Åbo Akademi University, Artillerigatan 6A, Turku 20520, Finland; Email: lisa.spoof@abo.fi (L.E.M.S.); jussi.meriluoto@abo.fi (J.A.O.M.); 2 Cell Biology, Department of Biosciences, Åbo Akademi University, Artillerigatan 6A, Turku 20520, Finland; Email: julia.misiorek@abo.fi (J.O.M.); diana.toivola@abo.fi (D.M.T.)

**Keywords:** cyanobacteria, toxins, microcystin, MC-LW, MC-LF

## Abstract

Microcystins (MC), cyanobacterial peptide hepatotoxins, comprise more than 100 different variants. They are rather polar molecules but some variants contain hydrophobic amino acid residues in the highly variable parts of the molecule. In MC-LF and MC-LW, the more hydrophobic phenylalanine (F) and tryptophan (W), respectively, have replaced arginine (R) in MC-LR. Depending on the structure, microcystins are expected to have different *in vivo* toxicity and bioavailability, but only a few studies have considered the toxic properties of the more hydrophobic variants. The present study shows that MC-LF and MC-LW have more pronounced cytotoxic effects on Caco-2 cells as compared to those of MC-LR. Treatment of Caco-2 cells with MC-LW and especially MC-LF showed clear apoptotic features including shrinkage and blebbing, and the cell–cell adhesion was lost. An obvious reduction of cell proliferation and viability, assessed as the activity of mitochondrial dehydrogenases, was observed with MC-LF, followed by MC-LW and MC-LR. Cytotoxicity was quantified by measuring lactate dehydrogenase leakage. The more hydrophobic MC-LW and MC-LF induced markedly enhanced lactate dehydrogenase leakage compared to controls and MC-LR, indicating that the plasma membrane was damaged. All of the three toxins examined inhibited protein phosphatase 1, with MC-LF and MC-LW to a weaker extent compared to MC-LR. The higher toxic potential of the more hydrophobic microcystins could not be explained by the biophysical experiments performed. Taken together, our data show that the more hydrophobic microcystin variants induce higher toxicity in Caco-2 cells.

## 1. Introduction

Some cyanobacterial species, such as those among the freshwater genera *Microcystis*, *Planktothrix* and *Anabaena*, produce secondary metabolites called microcystins [1]. These cyclic heptapeptides hepatotoxins are frequently reported worldwide and pose a threat to human health. 

Microcystins consist of more than 100 different variants with molecular weights of 900–1100 Da [2,3,4]. The general structure of microcystins is cyclo(-D-Ala-L-X-D-erythro-β-methylAsp(iso-linkage)-L-*Z*-Adda-D-Glu(iso-linkage)-*N*-methyldehydro-Ala) where Adda stands for 3-amino-9-methoxy-2,6,8-trimethyl-10-phenyldeca-4*E*,6*E*-dienoic acid, a unique β-amino acid [5,6,7,8]. Microcystins have a two-letter suffix nomenclature according to the amino acids at the highly variable positions 2 (X) and 4 (Z). For example, the highly toxic microcystin-LR (MC-LR) contains leucine at position 2 and arginine at position 4. Toxicological experiments have been conducted mostly with MC-LR and this has resulted in a guideline value. The provisional guideline value of MC-LR in drinking water, *i.e.*, the concentration of MC-LR that does not result in any significant risk to health during a lifetime exposure, set by World Health Organization, is 1 µg/L [9]. However, structural variations occur in all seven amino acid residues giving different variants distinctive properties [2]. When different microcystin variants have been evaluated and compared, differences regarding their toxicity have been shown [10,11,12,13]. Due to insufficient data, no WHO-guideline value has been assessed for any other microcystin variant. Commonly occurring microcystins in the environment are MC-LR, MC-RR, MC-YR and desmethylated variants of MC-LR and MC-RR [14,15]. MC-LW and MC-LF been reported less often, but when they were reported, the concentrations found have been quite high [15,16,17]. Since microcystins have been associated with both genotoxic and carcinogenic effects, the risks regarding this family of toxins should be thoroughly evaluated [18]. Microcystins disturb the reversible phosphorylation in cells by inhibiting protein phosphatases 1 and 2A through noncovalent and covalent binding to the enzymes [19,20,21,22,23]. Inhibition of protein phosphatases in mammals has been considered to be the key toxicity mechanism but other cell targets that have been suggested are the aldehyde dehydrogenase II [24] and the β subunit of ATP-synthase [25]. There are numerous bioactive peptides, among them microcystins, that need to be taken up by transport proteins or penetrate the cell membrane in order to elicit their action. Microcystins are actively transported into cells through members of the organic anion transporting polypeptides [26]. The way microcystins enter the organism, organs and cells has a crucial effect on the distribution of microcystins and their toxicity *in vivo*. Oral exposure to microcystins, through ingestion of contaminated water or food, has been considered the exposure route of highest concern to human health [1]. Considering ingestion as a major exposure pathway, cell lines like Caco-2 cells that resemble the small intestine are appropriate for studying cellular effects of microcystins [27].

Due to the presence of both hydrophobic and hydrophilic amino acids in these biologically active peptides, we decided to take a closer look at their first contact with the cell membrane. The ability of a compound to interact with biological membranes might influence the compound’s bioactivity, as well as pharmacological parameters like absorption, biotransformation, half-life and excretion of the compound [28]. Interactions of small molecules with biological membranes are difficult to study due to the complexity and heterogeneity of the membrane [29]. As an experimental model, bilayer vesicles of a few phospholipids were therefore used. We have previously shown that the amphipathicity of MC-LF and MC-LW enabled the more hydrophobic toxin variants to associate with lipids in monolayer experiments to a larger extent compared to MC-LR [30]. In MC-LF and MC-LW, the more hydrophobic amino acids phenylalanine (F) and tryptophan (W), respectively, have replaced arginine (R) in MC-LR. Encouraged by our earlier biophysical experiments [30], we continued to use model membranes as a first attempt to gain further insight into membrane interactions of microcystins. In addition, we examined cell morphology, cell toxicity, cell proliferation and protein phosphatase inhibition of MC-LR, MC-LW and MC-LF on a Caco-2 cell line. 

## 2. Results and Discussion

### 2.1. Microcystins Did Not Have an Effect on the Phospholipid Phase State, as Revealed by the Fluorescent Probe Laurdan

To study whether microcystins act already at the membrane level, several biophysical approaches are available. With the purpose to study whether the physical order of a vesicle bilayer is changed by microcystins, Laurdan was chosen as the fluorescent probe. Laurdan is equally distributed in the liquid ordered and the liquid disordered phases that coexist in DPPC vesicles at 40 °C [31]. When incorporated in vesicle membranes, Laurdan can reflect the relaxation rate of water molecules present at the interfacial region of the membrane bilayer. This can be indicated by calculating the generalized polarization (GP) value for Laurdan and by analyzing the emission spectra in the presence and absence of microcystins. However, the GP values were not influenced and the relative proportion of the two phases was not shifted in the presence of microcystins. No shift in the emission profile and no change in emission intensity were observed. 

Living cells are very complex entities and different kinds of models can be used to mimic biological membranes. Vesicle models are simpler than the biological membrane and a single, well-studied phospholipid, DPPC, was preferred here due to its appropriate characteristics. DPPC bilayers have, at the chosen temperature (40 °C), two coexisting phases, liquid ordered and liquid disordered phases. The high temperature increased the lateral and rotational motions of the lipid molecules and decreased the order of the hydrocarbon chains in the DPPC bilayer [32]. If some of the phases had been stabilized by the addition of a microcystin it could have been indicated by the fluorescence emission profile of the incorporated fluorophore Laurdan since the spectral properties of Laurdan are altered in response to lipid packing [33]. Laurdan is equally distributed in the ordered and disordered lipid phases and senses the orientation of water molecules present at the lipid interface [31]. Any reorientation of the solvent dipoles is connected to the penetration of water molecules into the lipid bilayer [34]. None of the microcystin variants, added externally to the DPPC vesicles, had any observable effect on the phase behavior of the bilayer. 

### 2.2. No Resonance Energy Transfer between MC-LW and the Fluorescent Probe Cholestatrienol

Resonance energy transfer can be used to measure distances between donor and acceptor molecules [35]. The tryptophan in MC-LW was used as the intrinsic fluorescent donor since it is able to transfer energy in its excited state to another chromophore [36]. This transfer of energy may occur depending, for example, on the orientation of the molecules and their distance from each other [37]. Cholestatrienol (CTL), a fluorescent analog of cholesterol, was used as an acceptor due to its suitable fluorescence properties [38]. Resonance energy transfer can occur if there is a spectral overlap of the emission spectrum of the donor with the absorption spectrum of the acceptor, provided that certain other criteria are met, such as the distance between the two molecules [35]. Here we tried to elucidate the location of tryptophan in MC-LW in relation to an incorporated sterol, CTL, in bilayers. The tryptophan in MC-LW acts as a donor and the fluorescent probe CTL located in the membrane interface as an acceptor. The emission intensities measured at 374 nm after excitation at 290 nm did not indicate any rate of energy transfer. MC-LW was not able to interact with CTL. Even though we have a good overlap between the emission spectrum of tryptophan and the absorption spectra of cholestatrienol, we could not see any increase in the emission intensity of the acceptor and concluded that energy transfer between the two fluorophores is not likely to occur. 

### 2.3. Dramatic Morphological Effects Caused by MC-LF and MC-LW

**Figure 1:**
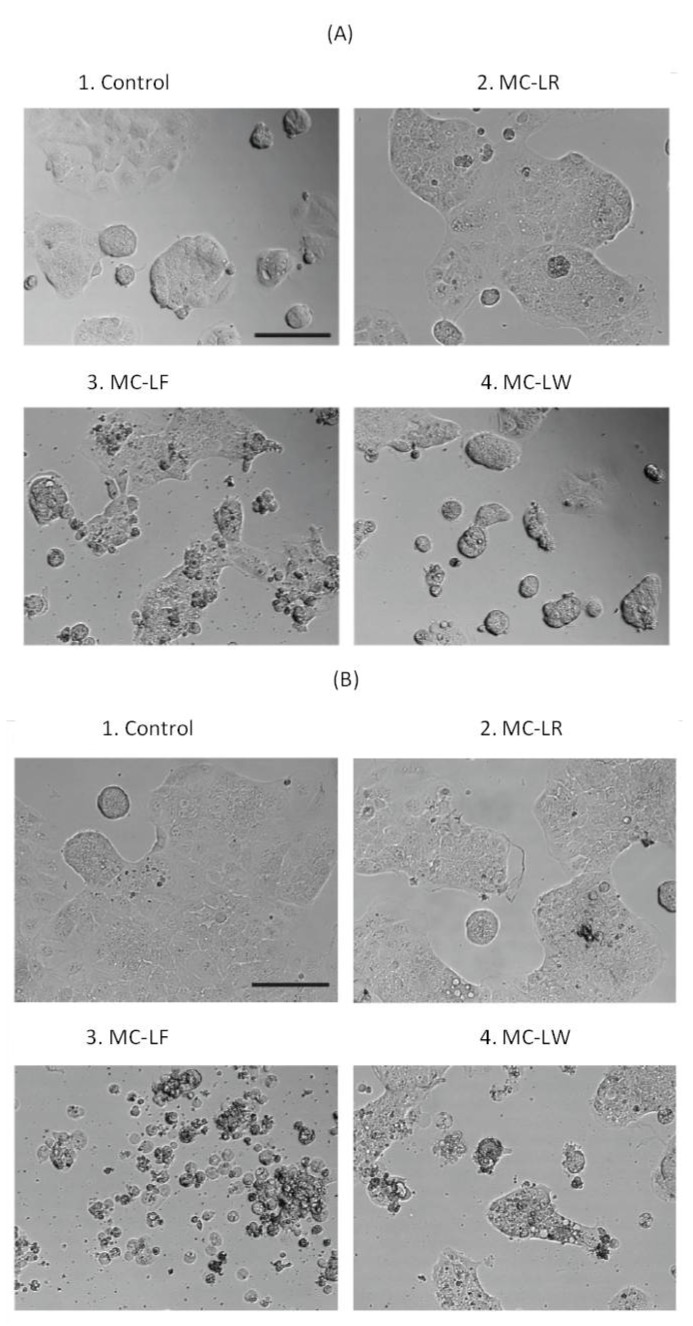
Morphological effects of MC-LW and MC-LF in Caco-2 cells. Phase contrast microscopy images show control cells were treated with PBS (**1**); Caco-2 cells after 50 µM MC-LR (**2**); MC-LW (**3**) and MC-LF (**4**) treatment for (**A**) 22 h and (**B**) 44 h. Scale bar is 100 µm.

Since MC-LR can enter hepatocytes and cause morphological alterations [39], we investigated if the three microcystin variants studied here could induce morphological changes in Caco-2 cells as an indication of membrane activity. Cells exposed to MC-LR (50 µM) did not show significant change in cell morphology, neither at 22 h (Figure 1A) nor 44 h (Figure 1B) after treatment, and resembled unaffected control cells (Figure 1A1,B1). Cells treated with MC-LW and especially MC-LF, however, showed clearly morphological alterations including apoptotic features with shrinkage, blebbing and loss of cell contact (Figure 1A,B) which appeared in a time (Figure 1A,B) and dose- (not shown) dependent manner. These results suggest that MC-LF and MC-LW have clear cellular effects compared to MC-LR. 

Microcystins initiate apoptosis and typical morphological changes in liver cells, as well as in several other cell lines, include membrane blebbing, shrinkage of cells, condensation of the chromatine and cytoskeletal reorganization [39,40,41,42,43]. Morphological examination of Caco-2 cells exposed to microcystins revealed remarkable differences between different microcystin analogs. MC-LW and especially MC-LF exposures caused severe anomalies in cell morphology. The spreading of the cells was changed by all three toxin variants with minor effects by MC-LR, and a severe loss of cell number and cell-cell adhesion by MC-LF. Blebbing, moreover, was particularly evident for MC-LF. 

### 2.4. MC-LF and MC-LW Suppress Caco-2 Cell Proliferation

**Figure 2:**
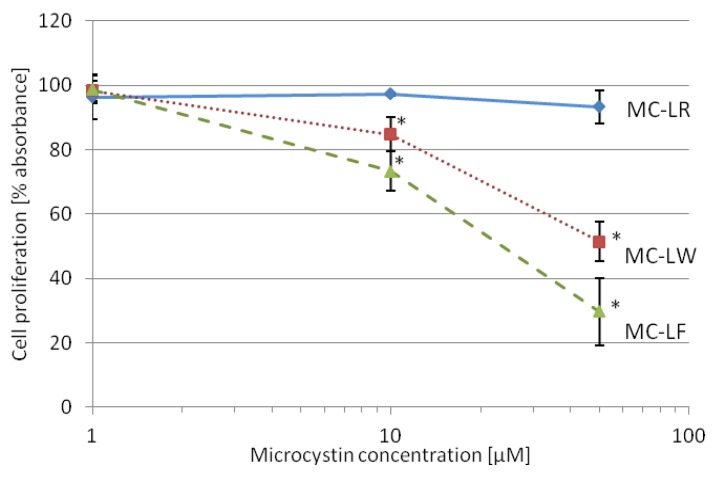
Effect of microcystin variants on Caco-2 cell proliferation, assayed by the WST-1 test. Caco-2 cells exposed to 1 µM, 10 µM and 50 µM of MC-LR (**―)**, MC-LW (**∙∙∙**) and MC-LF (---). On the y-axis the amount of formazan dye formed, which correlates to metabolically active cells, compared to control cells. Data shown are means of three individually performed experiments with three replicas each 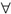 standard deviation. Values marked with * are statistically different from control cells (Student’s *t*-Test, *p* ≤ 0.05).

Next, the metabolic activity of cells, reflecting cell proliferation and cell viability, was measured using the WST-1 assay. The tetrazolium salt WST-1 is cleaved to the dark red formazan in a reaction catalyzed by mitochondrial dehydrogenases [44]. An expansion of viable and metabolically active cells leads to increased mitrochondrial dehydrogenases and increased cleavage of formazan. A toxin concentration of 50 µM reduced the activity of mitochondrial dehydrogenases to 93% for MC-LR, 51% for MC-LW and 30% for MC-LF after 48-h toxin exposure (Figure 2) compared to the control. At 10 µM toxin the activity was also reduced but to a lesser extent, to 97% for MC-LR, 84% for MC-LW and 73% for MC-LF (Figure 2), compared to the control. At 1 µM, cell proliferation did not show a statistically significant decrease by any of the microcystins tested (Figure 2). These data show that the more hydrophobic microcystin variants MC-LF and MC-LW inhibit Caco-2 cell proliferation in a larger extent than MC-LR. 

### 2.5. MC-LF and MC-LW Induced Caco-2 Cell Death

**Figure 3:**
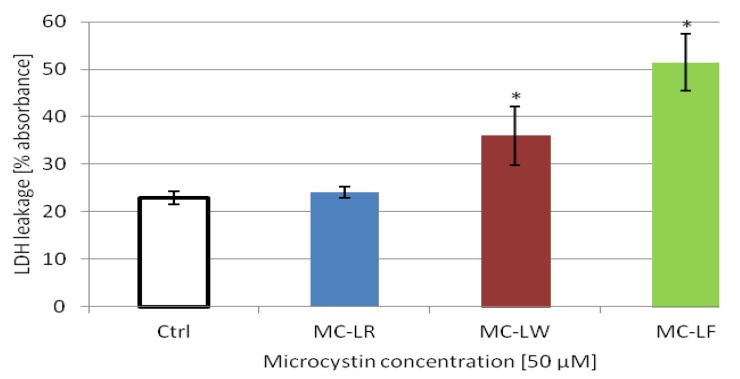
Effect of microcystin variants on Caco-2 cell toxicity, assayed by LDH leakage. Caco-2 cells were exposed for 48 h to 50 µM of microcystin-LR, microcystin-LW and microcystin-LF and assayed for LDH leakage. The % LDH leakage from cells treated with toxins was calculated from the maximum cellular LDH leakage. Data shown are means from three individually performed experiments with three replicas each. Values marked with * are statistically different from control cells (Student’s *t*-Test, *p* ≤ 0.05).

Leakage of LDH was measured from Caco-2 cells treated with 50 µM toxins for 48 h to quantify plasma membrane damage. Lactate dehydrogenase is a stable cytoplasmic enzyme present in all cells. When the plasma membrane is damaged, LDH is rapidly released into the cell culture supernatant [45]. Caco-2 cells not treated with toxins had the same release of LDH that MC-LR, about 25% of total (Figure 3). Cells treated with MC-LW and MC-LF released more LDH, 36% and 51%, respectively, which were statistically significant (*p* ≤ 0.05, *t*-test) from controls and MC-LR treated Caco-2 cells. The % LDH-release from cells treated with toxins was calculated from the maximum cellular LDH released by lysing the cells. Taken together, these data clearly show that the three microcystins variants studied induced increased cell death (Figure 2) and increased morphological alterations (Figure 1) that are higher in the more hydrophobic variants, MC-LF > MC-LW > MC-LR. 

The LD_50_ values of microcystins (mouse, i.p.) are usually within the range of 43–600 µg/kg [2,10]. Recently it has been shown that in primary hepatocytes the rank order of toxicity was MC-YR > nodularin > MC-LR [46]. Moreover, the desmethylated variants tested showed a higher toxicity than their fully methylated counterparts [46]. However, toxicological data for several microcystin variants, e.g. MC-LW and MC-LF, are scarce. Microcystins are believed to target mainly the liver since hepatocytes have several types of organic anion transporters that actively take up the toxins [11,26]. Different cell lines, serving as models for the corresponding cell types, as well as *in vivo* experiments, have been used to study whether also other organs and tissues might be affected by microcystins. The kidneys show *in vitro* and *in vivo* effects and are considered to be target organs for microcystins [47,48,49,50]. Cells of the intestine and the colon were shown to be affected by microcystins [51,52] as well as cells representing the heart, brain and immune system [26,53,54]. Several other cell lines have also been tested [55,56]. The toxicity studies are complicated to compare since the toxin concentrations vary, as well as the material of origin, the experimental setup and the endpoints used. Caco-2 cells treated with pure MC-LR and MC-LR containing cyanobacterial extract have shown high alterations with several oxidative stress biomarkers [57]. Caco-2 cells are human colon adenocarcinoma cells widely used for drug permeability and drug metabolism screening [58]. They are easy to culture, are robust and give a good experimental reproducibility [59]. The present study shows that certain microcystin variants, MC-LW and MC-LF, have a more pronounced cytotoxic effect on the number of Caco-2 cells. Metabolically active cells diminished considerably, measured by formazan cleavage already at a concentration of 10 µM of MC-LF and MC-LW. The suppression of mitochondrial dehydrogenase activity of MC-LF and MC-LW might enhance apoptosis in Caco-2 cells. Both analogs also showed an increase in LDH activity indicating a loss of plasma membrane integrity of the cells exposed. Culture conditions for Caco-2 cells have a remarkable effect on both morphology and the carrier-mediated transporters of the cells [60]. In our experimental setup, we were not able to see the striking effects of MC-LR on Caco-2 cells reported by Botha *et al.* [61]. In our experiments a higher concentration of cells were used and the toxins were added at a time when cells had already formed a stable monolayer, *i.e.* one day after seeding. However, the obvious effect of both MC-LW and MC-LF is in agreement with previous results, where it was concluded that the more hydrophobic microcystin variants were clearly more toxic than MC-LR *in vitro* and thus potentially also *in vivo* [11,62]. 

### 2.6. MC-LF and MC-LW Were Weaker Protein Phosphatase Inhibitors

**Figure 4:**
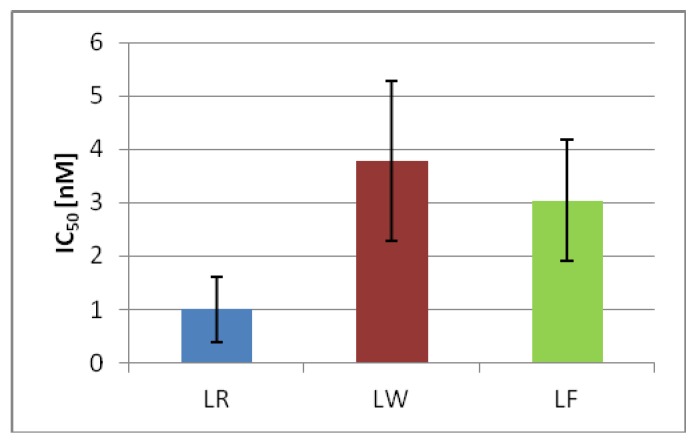
Protein phosphatase 1A inhibition of microcystin variants expressed as IC50 (nM) in the PP1A inhibition assay. Values are means of three individually performed experiments, each in duplicate.

Since MC-LR has been shown to act through protein phosphatase inhibition, the effect of microcystin variants were compared with respect to their capacity to inhibit PP1A. Data extracted from standard curves for the inhibition of protein phosphatases show that PP1A was inhibited by all three toxins. MC-LF and MC-LW inhibited protein phosphatases to a lesser extent than MC-LR. The IC_50_ value (nM) was 3.0 for MC-LF and 3.8 for MC-LW, compared to 1.0 for MC-LR (Figure 4). 

Inside the cells microcystins interact with protein phosphatases, mainly PP1 and PP2A, and inhibits their action by a two-step mechanism [63]. The amino acid *N*-methyldehydroalanine forms a stable covalent bond to Cys273 in PP1 and Cys266 in PP2A, after a first rapid noncovalent binding step [21,22,64]. The conformation of MC-LR allows the molecule to bind with high affinity to PP1. The long hydrophobic Adda side chain fits well into a hydrophobic groove in PP1, and the leucine side chain packs closely to Tyr272 near the active site [65]. The conformation of MC-LR in complex with PP1 is very similar to the conformation of MC-LR in solution which probably contributes to the fact that MC-LR is a strong inhibitor of PP1 [65]. Conformational changes in the microcystin molecule will probably affect the protein phosphatase inhibition capacity. Enlarging the hydrophobic part of the molecule with tryptophan (MC-LW) or phenylalanine (MC-LF) might not allow Adda to fit smoothly into the pocket in the enzyme. MC-LR appears to be the strongest inhibitor of the phosphatases among the studied microcystins [11,19,62]

Here, as in Blom and Juttner (2005), the protein phosphatase inhibition and the acute toxicity did not correlate with each other since the more hydrophobic analogs were weaker inhibitors but showed more pronounced cytotoxic effects [66]. However, the toxic effect of MC-LW and MC-LF could be due to a higher entrance of MC-LW and MC-LF into the Caco-2 cells, facilitated by an OATP member perhaps due to, for instance, a higher membrane activity.

## 3. Experimental Section

### 3.1. Reagents

Reversed-phase high performance liquid chromatography of *Microcystis* PCC7820 extracts (Pasteur Culture Collection, Paris, France) was used to purify (>98%) and quantify MC-LR, MC-LF and MC-LW [67]. The toxins were dissolved in phosphate buffered saline (PBS; 137 mM NaCl, 2.7 mM KCl, Na_2_HPO_4_H_2_H_2_O 10 mM, KH_2_PO_4_ 1.76 mM; pH 7,4). 1,2-Dipalmitoyl-sn-glycero-3-phosphocholine (DPPC) and 1-palmitoyl-2-oleoyl-sn-glycero-3-phosphocholine (POPC) was purchased from Avanti Polar Lipids, Inc. 6-dodecanoyl-2-dimethylaminonaphtalene (Laurdan) was obtained from Molecular Probes (Leiden, The Netherlands). Cholesta-5,7,9(11)-trien-3-beta-ol (CTL) was synthesized according to the method described by Fischer *et al*. [68] and purified using reversed-phase HPLC [69]. Protein phosphatase 1 was isolated from *E. coli* expressing rabbit skeletal muscle PP1 (New England Biolabs, MA, USA). All chemicals used were of analytical or chromatographic grade.

### 3.2. Generalized Polarization

Lipid vesicles were prepared of DPPC in a buffer containing 20 mM Tris-HCl and 145 mM NaCl (pH 7.0). After 2 min of sonication (duty cycle 40%, output control 5) with a Branson probe sonifier W-250 (Branson Ultrasonics, Danbury, CT, USA), unilamellar vesicles were made by repeated extrusion using a Lipextruder (Lipex Biomembranes, Vancouver, BC, Canada) through 100 nm polycarbonate filters (Costar Corp., Cambridge, MA, USA), according to Hope *et al*. [70]. Laurdan (final concentration of 1 mol%) was dissolved in ethanol and incorporated to the vesicles by shaking for 30 min. Laurdan emission spectra was recorded between 400 and 580 nm with a PTI QuantaMaster 1 spectrofluorimeter (Photon Technology International, Lawrenceville, NJ, USA), after excitation at 365 nm. The temperature (40 °C) was controlled by a Peltier element with a temperature probe submerged in the sample. The generalized polarization (GP) was calculated according to


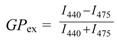


where *I*_440_ is the fluorescence intensity at 440 nm, which is the characteristic wavelength of the liquid disordered phase, and *I*_475_ is the emission intensity at 475 nm, the characteristic wavelength of the liquid ordered phase [33]. The emission spectra of Laurdan was measured and compared in the absence and presence of toxins (1 and 10 µM final concentration). Tamoxifen was successfully used as a positive control [71].

### 3.3. Fluorescence Resonance Energy Transfer

Lipid vesicles were prepared of POPC and CTL (molar ration 99:1) by probe sonication. The phospholipid was dried under argon at 40 °C with excess solvent removed by vacuum drying for 20 min, and resuspended in argon-purged Tris-HCl buffer. After addition of CTL the lipids were briefly vortexed and sonicated (duty cycle 20%, output control 5) for 2 min using a Branson W-250 probe sonifier (Branson Ultrasonics, MA, USA). The fluorescence emission intensity of CTL was measured at 374 nm after excitation at 290 nm using Photon Technology International (Lawrenceville, NJ, USA). The fluorescence emission intensity was compared in the absence and presence of toxins (5 µM final concentration). 

### 3.4. Caco-2 Cells

Caco-2 cells (human epithelial colorectal adenocarcinoma cells, DSMZ, Braunschweig, Germany) were maintained at 37 °C in a humidified 5% CO_2_/95% O_2_ atmosphere in plastic dishes in Dulbecco’s modified Eagles’ medium supplemented with 10% Fetal Calf Serum, 1% L-glutamine, 100 U/mL penicillin and 100 µg/mL streptomycin. Culture media and supplements were purchased from Sigma. For cell proliferation and cytotoxicity assays cells were seeded (100 µL) the day before the start of the experiment in 96-well microtiter plates (BD Falcon, Helsinki, Finland) at densities of 80,000 cells/mL. For this, cells were trypsinized and counted using a cell haemocytometer (Neubauer improved, Marienfeld, Germany) and trypan blue. The cells were washed and fresh media (90 µL) was added to all wells when the experiments were started. For experiments, cells were treated with 5 µL PBS (control cells) or 5 µL of each toxin dissolved in PBS (final toxin concentration 1, 10 or 50 µM) at least in triplicates.

### 3.5. Cell Morphology

To determine the effects of microcystins on Caco-2 cell morphology, cells were exposed to the different microcystins at 1, 10 and 50 µM for 22 h and 44 h, and photographed under phase contrast in a Leica DMIL light microscope using a Leica EC3 digital camera. Images were compiled using Adobe Photoshop and Illustrator. 

### 3.6. Cell Proliferation

To study the effects of microcystins on Caco-2 cell proliferation, the Cell Proliferation Reagent WST-1 (Roche Applied Sciences, Mannheim, Germany) was used. Cells were treated for 48 h in 96-well microtiter plates with MC-LR, MC-LW and MC-LF (0, 1, 10 and 50 µM) and the assay was performed according to the manufacturer’s suggestions. Briefly, the amount of formazan dye formed was measured on a multiwall spectrophotometer (Varioskan Flash, Thermo Fisher Scientific Inc., Vantaa, Finland) at 450 nm after the addition of 10 µL of Cell Proliferation Reagent WST-1 to cells treated with or without MC-variants and control. 

### 3.7. Cytotoxicity Assay

Membrane damaging effects were estimated by lactate dehydrogenase (LDH) leakage using a Cytotoxicity Detection Kit (Roche Applied Sciences, Mannheim, Germany). The test was performed according to the LDH assay protocols. During treatment the percentage of FCS did not exceed 1%. Briefly, a reaction mixture was added to all wells to monitor the leakage of LDH into the extracellular fluid. A lysis solution was added to wells for the maximum cellular amount of LDH. Cells not treated with toxins reflect the spontaneous baseline leakage of LDH. The % LDH leakage from cells treated with 0, 1, 10 and 50 µM MC-LR, MC-LW and MC-LF for 48 h was calculated from the maximum cellular LDH leakage. 

### 3.8. Protein Phosphatase Inhibition

Inhibition of protein phosphatases was analyzed by a colorimetric protein phosphatase inhibition assay protocol similar to that described by An and Carmichael [72]. Samples and calibrators (0.125–4 µg/L) were dissolved in water and 10 µL PP1 (activity of 1.67 U/mL, dissolved in buffer) was added to each microtiter plate well, except to the negative control. After addition of substrate (15 mM p-NPP), a two-hour incubation at 37 °C followed and absorbance was measured at 405 nm on a 1420 Victor multilabel counter (PerkinElmer, Turku, Finland). Samples, calibrators and negative control were performed in duplicates and the assay was repeated three times. The inhibition percentages were calculated as: average OD of calibrator 0–average OD of standard or sample × 100/average OD of standard. IC_50_, expressed in nM, was calculated as the concentration of toxins that inhibited the release of PP by 50% compared with the uninhibited control reaction. 

## 4. Conclusions

We conclude that the toxic potential of different microcystin analogs showed clear differences in Caco-2 cells, with MC-LF being most toxic, closely followed by MC-LW. When evaluating the risk associated with cyanobacterial blooms, the morphological examination, the cytotoxicity and the cell viability tests, and the protein phosphatase inhibition capacities discussed here, all emphasize the need to pay attention to which microcystin analogs are occurring. The biophysical experiments performed in this study did not demonstrate that solely the membrane effects of the more hydrophobic microcystins would have a facilitated entrance into cells without the aid of cellular components, such as transporters. 
